# Blockade of p75 Neurotrophin Receptor Reverses Irritability and Anxiety-Related Behaviors in a Rat Model of Status Epilepticus

**DOI:** 10.22034/ibj.22.4.264

**Published:** 2018-07

**Authors:** Soraya Mehrabi, Mahyar Janahamdi, Mohammad Taghi Joghataie, Mahmood Barati, Mohsen Marzban, Mahmoudreza Hadjighassem, Maryam Farahmandfar

**Affiliations:** 1 Department of Neuroscience, School of Advanced Technologies in Medicine, Tehran University of Medical Sciences, Tehran, Iran; 2 Neuroscience Research Center and Department of Physiology, School of Medicine, Shahid Beheshti University of Medical Sciences, Tehran, Iran; 3 Department of pharmaceutical biotechnology, School of pharmacy, Shahid Beheshti University of Medical Sciences, Tehran, Iran; 4 Cellular and Molecular Research Center, Iran University of Medical Sciences, Tehran, Iran; 5 Department of Neuroscience, Faculty of Advanced Technologies in Medicine, Iran University of Medical Sciences, Tehran, Iran; 6 Brain and Spinal Cord Injury Research Center, Neuroscience Institute, Tehran University of Medical Sciences, Tehran, Iran

**Keywords:** Anxiety, Pilocarpine, Status epilepticus

## Abstract

**Background:**

Many recent epidemiological studies have shown that epileptic patients are more likely suffer from depression, anxiety, and irritability. However, the cellular mechanisms of epilepsy-induced psychotic behaviors are not fully elucidated. Neurotrophin receptors have been suggested to be involved in epilepsy and also in psychiatric disorders. Up-regulation of p75NTR expression and activation of p75NTR signalling cascades after the seizure have been shown, but the role of the p75 receptor in epilepsy-induced psychotic behaviors has not been documented so far. Therefore, the present work aimed to investigate the effect of p75 receptor blockade on seizure activity, irritability, and anxiety-like behaviors in a rat model of status epilepticus.

**Methods:**

Rats were injected with pilocarpine (350 mg/ kg, i.p.) to induce status epilepticus. Then various behavioral tests were performed after the blockade of p75NTR alone or in combination with p75 antagonist and phenobarbital. Molecular analysis by PCR was performed to investigate the expression of p75 and pro-NGF.

**Results:**

Molecular findings indicated a high level of mRNA expression for both p75 receptors and pro-NGF in the epileptic model group. Results also showed that the administration of p75 antagonist alone or in combination with phenobarbital was able to significantly influence the behavioral responses. Furthermore, 20-hours video monitoring showed a decrease in the frequency and duration of seizures in the rat group receiving p75 antagonist.

**Conclusion:**

Taken together, the present study suggests that the blockade of the p75 receptor may affect the irritability and anxiety-related behavior in a rat model of status epilepticus.

## INTRODUCTION

EPilepsy is one of the most common neurological disorders with a prevalence of 1% in the general population. This disorder is associated with the excessive electrical discharges in the neurons. Studies have shown that the prevalence of psychiatric disorders in patients with epilepsy is more frequent than that in general population[1,2]. More than one in four epileptic patients have experienced psychological problems, which might need treatment, and more than 10% of patients in psychiatric hospitals are diagnosed with epilepsy[3]. Seizure is related with neurological and behavioral problems such as anxiety, memory impairment, psychosis, and cognitive dysfunction, which affect the quality of life of patients[4-6].

Several lines of evidence have indicated that nerve damage and sclerosis occur in different areas of the brain, especially in the hippocampus of patients with temporal lobe epilepsy (TLE)[7-10]. Thus, after the initial damages, this area could be considered as the origin of spontaneous seizures. In addition, since the hippocampus, as a part of the limbic system, plays an important role in learning and memory and mood stability, its destruction can cause behavioral abnormalities such as fear, anxiety, depression, memory impairment, and hyperexcitability[11]. In TLE, hyperexcitability and neuronal loss in hippocampus evoke psychiatric problems, such as mood disturbances. On the other hand, hippocampal atrophy and synaptic disturbances, which occur in depression, increase neuronal excitability, thereby leading to TLE[12]. Therefore, epilepsy and neuropsychiatric disorders may have common pathophysiological mechanisms.

The involvement of neurotrophins in several nervous system disorders, including epilepsy and neuropsychiatric disorders, has been reported[13,14]. Neurotropic factors have been considered as important candidates for therapeutic intervention against neurological diseases. Considering the important role of these factors in the nervous system, investigation of their signalling pathways and ligand-receptor interactions is essential. One of these signalling pathways is related to the function of p75 neurotrophin receptors, which is a transmembrane receptor for tumor necrosis family of growth factors, including nerve growth factor (NGF), brain-derived neurotrophic factor (BDNF), neurotropic 3 (NT3) and neurotropic 4 (NT4)[15,16] .

Previous studies have reported that high affinity of pro-neurotrophins to p75 receptors increases the rate of neuropsychiatric diseases including depression[17]. These receptors are widely expressed throughout the brain in embryonic periods. Hippocampal neurones also express p75 receptors in various stages of development[18]. Expression of the p75 receptor is increased in the hippocampus after seizures as well as in the forebrain cholinergic neurons following damages due to the excessive excitability of these neurons. In addition to this, re-expression of p75 in the aforementioned cells may be a part of damage-induced plasticity, which will start the mechanism of evolutionary recapitulation in the case of lesions and stressors, leading to the production of pro-NGR in some brain areas where p75-dependent cell death may occur[19-23].

p75 re-expression has been indicated to be a part of a homeostatic program to destroy neurons, axons, and synapses in the degenerated or damaged areas[24]. Therefore, it seems quite logical that the p75 signalling pathway can be regarded as a therapeutic target for these conditions and has to be taken into consideration. The reason for this consideration might be that epilepsy comorbidity with the most psychiatric disorders in epileptic patients is struggling with these issues[17,25]. This comorbidity has still not been well defined, and the relationship between epilepsy and psychiatric disorders has not been fully determined although some attempts have been made to find a correlation between epilepsy and mental health problems[26]. Various animal models have been developed for the study of epilepsy, and among them, pilocarpine-induced epilepsy is one of the most common models in the rodent. In this model of epilepsy, development of spontaneous seizure results in behavioral disturbances, which may take three weeks up to three months after pilocarpine-induced status epilepticus. Investigations performed on the pilocarpine model have reported that epilepsy can be divided into three stages: acute, latent, and chronic[27,28]. However in two recent studies, this concept was changed, so that in the first days after induction of seizures, epilepsy was divided into the immature and mature phases, where the onset of aggression and agitation in behavior coincided with the mature phase of seizures[29,30].

The present study, by using molecular and behavioral approaches, aimed to answer two questions: The first one is what would be the behavioral consequences of enhancement of p75 and pro-NGF levels in the animal model of TLE induced by pilocarpine, and the second one is whether p75 receptor blockade can be effective on behavioral changes induced by pilocarpine injection.

## MATERIALS AND METHODS

### Animals

Adult male Wistar rats, provided by Shahid Beheshti University Animal House (Tehran, Iran), were housed under controlled standard conditions (12 h light/dark cycle), with food and water available *ad libitum* a week before. Rats were divided into three main groups (n = 7 rats in each group), including the control group, vehicle-treated group, and pilocarpine-treated group. The pilocarpine-treated group was further subdivided into four groups (pilo-treated alone, pilo-treated+p75 antagonist, pilo-treated + p75 antagonist + phenobarbital, and pilo-treated + pheno-barbital; n = 7 rats were assigned for each group. The dose of P75 antagonist was chosen based on previous studies[31,32]. All experiments were performed in accordance with the animal care and management rules approved by the Ethics Committee of Iran University of Medical Sciences (Tehran, Iran).

### Drugs and chemicals

Phenobarbital was purchased from Temad Co. (Tehran, Iran). Pilocarpine hydrochloride and scopolamine methyl nitrate were procured from Sigma-Aldrich (St. Louis, MO, USA). p75 neurotrophic factor antagonist peptide was synthesized and purchased from GenScript, USA, with a minimum 92.9% purity.

### Induction of pilocarpine model of epilepsy

To induce epilepsy, rats were injected with pilocarpine hydrochloride (380 mg/kg, i.p.; Sigma-Aldrich, UK), a muscarinic cholinergic agonist. In order to prevent the peripheral cholinergic effects of pilocarpine, rats were pre-treated with scopolamine methyl nitrate, as a cholinergic antagonist (1 mg/kg, i.p.; Sigma-Aldrich, UK) 30 minutes before the injection of a single dose of pilocarpine hydrochloride. Then the treated rats were monitored for a period of 3 to 4 hours after injection of pilocarpine, and the seizure severity was graded according to Racine’s criteria[33]. Only rats were included in the present work if they exhibited stage 4 or 5 seizure score. In order to terminate the sustained seizure that lasted for 3 hours, diazepam (7 mg/kg, i.p.) was injected, and an additional dose of diazepam (3 mg/kg, i.p.) was administered every 2 hours, if needed, and then the animals were cared and fed with Hartmann’s solution till recovery[34]. Two weeks after pilocarpine injection, the occurrence of spontaneous seizures was recorded during chronic phase using daily video monitoring 8 hours/day for two weeks.

### Behavioral assessment

Excitability and sensory responsiveness of control and pilocarpine-treated rats were evaluated using different behavioral tests, including approach-response, touch-response, finger-snap, and pick-up tests as described by Huang *et al*.[35]. Behavioral assessments were performed two weeks after status epilepticus induction between 9 a.m. and 12.30 p.m. Three groups of epileptic rats were randomly selected for i.p. injection of p75 antagonist alone (1 mg/kg dissolved in normal saline; n = 7) or an injection of phenobarbital alone (30 mg/kg, dissolved in normal saline; n = 7), or co-administration of p75 antagonist and phenobarbital with 30 minutes interval between injection of phenobarbital and p75 antagonist. All injections and tests were done for six days that were performed in the home cage with 30-min intervals between each test[36].

### Approach-response test

A pen held vertically was moved slowly toward the face of the animal, and responses were scored as 1, the rat had no reaction; 2, the rat sniffed at the pen; 3, the rat moved away from the pen; 4, the rat was frozen; 5, the rat jumped away from the pen; 6, the rat jumped at or attacked the pen.

### Touch-response test

The animal was gently prodded in the rump with the blunt end of a pen. Responses were recorded as 1, the rat has no reaction; 2, the rat turned toward the object; 3, the rat moved away from the object; 4, the rat frozen; 5, the rat turned toward the touch; 6, the rat turned away from the touch; 7, the rat jumped with or without vocalizations[35,37].

### Finger-snap test

A finger snap several inches above the head of the animal was performed. Responses were scored as 1, the rat had no reaction; 2, the rat jumped slightly (normal reaction); 3, the rat jumped suddenly[35,38].

### Pick-up test

The animal was picked up by grasping around the body. Responses were scored as 1, very easy; 2, easy with vocalizations; 3, some difficulties, the rat reared and faced the hand; 4, frozen (the rat); 5, difficult, the rat avoided the hand by moving away; 6, very difficult, the rat behaved defensively and may attacked the hand[35,37]. The behavioral tests were done by three independent observers, and the means of their scores were calculated for each animal for each test.

### Video monitoring of behavioral seizure

To evaluate the frequency and duration of spontaneous recurrent seizures (SRS), the rats were videotaped and then reviewed by an investigator blinded to treatment groups to score the number and cumulative duration of tonic-clonic seizures. Origin 7.5 SR6 (Microcal Software, Northampton, MA, USA) software was used for data recording and analysis. Power spectrum analysis was performed after applying a Hamming window function[39].

### Elevated plus maze

For assessing anxiety-related behavior and agitation, elevated plus maze test was performed in which maze was made of black-painted wood with four elevated arms raised by a single central support to a height of 62 cm above the floor. It was arranged as a cross with two open arms (45 × 10 cm) facing each other, and two other arms enclosed by high walls (45 × 10 × 40 cm). The four arms extended from a common central platform (10 × 10 cm). Ridges of 0.5 cm bordering the open arms were added to provide an additional grip. Behavior on the maze was recorded by a video camera mounted above the plus maze apparatus and connected to a monitor and a video tracking, motion analysis and behavior recognition system (EthoVision®, Noldus, Wageningen, Netherlands) in a room under low-intensity light (20 l×). The maze was divided into five areas, one for each arm and one for the centre (central platform). Programming and data recording equipment were controlled by a computer[40].

### Real-time RT-PCR analysis of mRNA expression

The mRNA level p75 and pro-NGF genes in hippocampal tissue were determined in different groups of rats (n = 4 for each group). The rats were sacrificed at one week, two weeks, and three weeks after the induction of TLE model by pilocarpine, and then hippocampal tissue was dissected over cold PBS. Briefly, total cellular mRNA was isolated using QIAzol, according to the manufacturer’s protocol. The quantity and quality of the isolated RNA was assessed by Spectrophotometery (Ultrospec 2000 UV/VIS spectrophotometer, Pharmacia Biotech, Freiburg, Germany). cDNA was generated from 1 µg of total RNA by reverse transcription using the cycle script reverse transcription system (Bioneer, Korea). The mRNA expression levels of p75NTR and pro-NGF and GAPDH were determined by quantitative real-time RT-PCR, using a Rotor-Gene 6000 Real-Time Thermal Cycler (Qiagen, Germany). GAPDH mRNA was used as the internal control. The PCR reactions were set up in a volume of 10 µL containing 1 µL cDNA, 5 µL AccuPower® 2X GreenStar qPCR Master Mix (Bioneer, Korea) and 10 pM of each forward and reverse specific primer. Reaction mode was set at 95 °C for 10 min, followed by 40 cycles of 95 °C for 15 s and 60 °C for 1 min. Amplification specificity was checked using verifying a single peak in melting curves. All samples and controls were normalized to the reference gene. No template controls and no reverse transcriptase control were included in each PCR run. All assays were carried out three times as independent PCR runs for each cDNA sample. The ΔΔCT method[41] was used to quantify the amplification-fold difference between groups; each gene expression was normalized with respect to GAPDH mRNA content. To validate the use of ΔΔCT method, a 5-fold serial dilution was performed on a cDNA sample over a 125-fold range. For each dilution sample, amplifications were performed in triplicate using reference and target gene primers. The average CT of all tests was calculated, and the ΔCT of target (p75NTR anf pro-NGF) and reference (GAPDH) genes were determined. A plot of the log cDNA dilution versus ΔCT (ΔCT target-ΔCT reference) was made for each target and reference genes, and the slope of fitted line was determined[39].

### Statistical analysis

All statistical analyses were performed using SSPS 19.0 (SPSS Inc., Chicago, IL, USA) and Prism software (version 6.0). Two-way analysis of variance (ANOVA) with Bonferroni’s post hoc’s test was used to examine significant differences between the groups. All results are presented as mean values ± standard error of mean (SEM), and *p* < 0.05 was considered as statistically significant difference.

## RESULTS

### Behavioral changes associated with status epilepticus

**Fig. 1:**
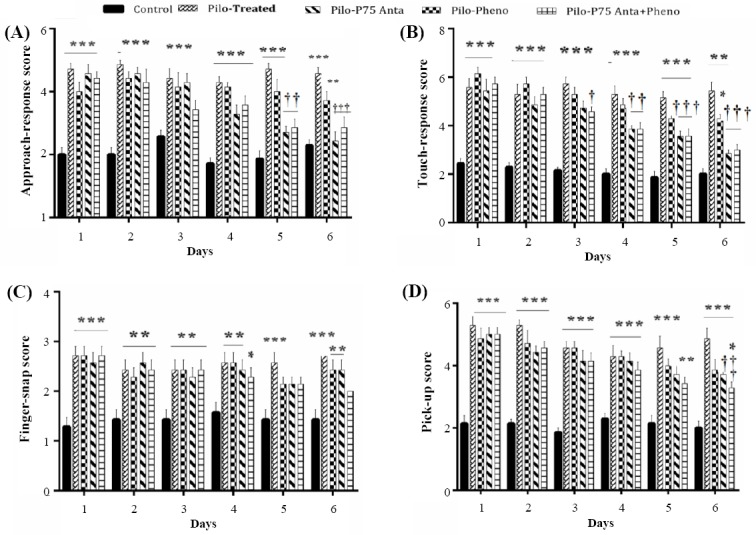
Behavioral assessment of P75NTR blockage in a rat model of pilocarpine-induced status epilepticus using approach-response (A), touch-response (B), finger-snap (C), and pick-up (D) on six days following SE induction. Bars represent mean ± SEM of the score. Two-way ANOVA was followed by post hoc comparisons using the Bonferroni’s method between control, pilocarpine-treated, pilo + P75 antagonist, pilo + phenobarbital, and pilo + P75 antagonist+phenobarbital. *represents significant difference between all treated groups and the control rats (^∗^*p* < 0.05, ^∗∗^*p* < 0.01, ^∗∗∗^*p* < 0.001) and † shows significant difference between pilocarpine-treated rats and the control and other treated animals (^†^*p* < 0.05, ^††^*p* < 0.01).

Since p75NTR is up-regulated following induction of SE[42] and may be involved in several psychiatric diseases[17,25]; therefore, we here asked whether the blockage of p75NTR would ameliorate behavioral changes associated with pilocarpine treatment in a rat model of SE. Findings showed that pilocarpine-treated rats exhibited significantly higher scores for aggression and anxiety in the approach and in the touch-response tests. In the approach-response test, the average score in control rats was 2.07 ± 0.21 versus 4.59 ± 0.19 in the pilocarpine-treated rats (*p* < 0.001; Fig. 1A). In the touch-response test, the average score for control rats was 2.11 ± 0.20 versus 5.40 ± 0.34 in the pilocarpine-treated rats (*p* < 0.001; Fig. 1B). Similarly, in the finger-snap test, epileptic rats were more disturbed, showing the higher levels of anxiety and restlessness (2.52 ± 0.19) as compared to the control rats (1.42 ± 0.19, *p* < 0.001; Fig. 1C). In the pick-up test, the mean score was significantly elevated in pilocarpine-treated rats (4.57 ± 0.36, *p* < 0.001) when compared to control rats (2.09 ± 0.20; Fig.1D). These results indicated that epileptic rats displayed significantly the increased levels of aggressive behavior compared to the control rats. Next, the effects of p75NTR antagonist treatment alone or in combination with phenobarbital on behavioral responses were investigated. Pilocarpine-treated rats were given daily i.p. injection of p75NTR antagonist alone, phenobarbital alone, or a combination of both for six days and then behavioral tests were performed over a period of six days, starting from the second weeks after the induction of SE. The results of the behavioral tests showed that treatment with p75NTR antagonist alone or with phenobarbital improved the behavioral responses in approach resulted in a significant lowered mean scores when compared with pilocarpine-treated alone rats during days 5-6 (*p* < 0.001; Fig. 1A). Blockade of p75NTR and treatment with combined p75NTR antagonist and phenobarbital led to a significant decrease in the touch-response score at 5-6 days (*p* < 0.001, Fig. 1B). Phenobarbital treatment alone did not affect the aggressive behavioral scores compared to the epileptic rats (Figs. 1A and 1B). Hypersensitive responses of epileptic rats were also evident both in the finger-snap and pick-up tests (Figs. 1C and 1D), and treatment with p75NTR antagonist alone or in combination with phenobarbital significantly reduced the behavioral hyperexcitability in epileptic rats on days 5 and 6 (*p* < 0.001; Figs. 1C and 1D). However, there were no significant differences between either p75NTR antagonist treatment alone or when co-treated with phenobarbital and control animals in all performed tests.

### Duration and frequency of spontaneous recurrent seizures

**Fig. 2:**
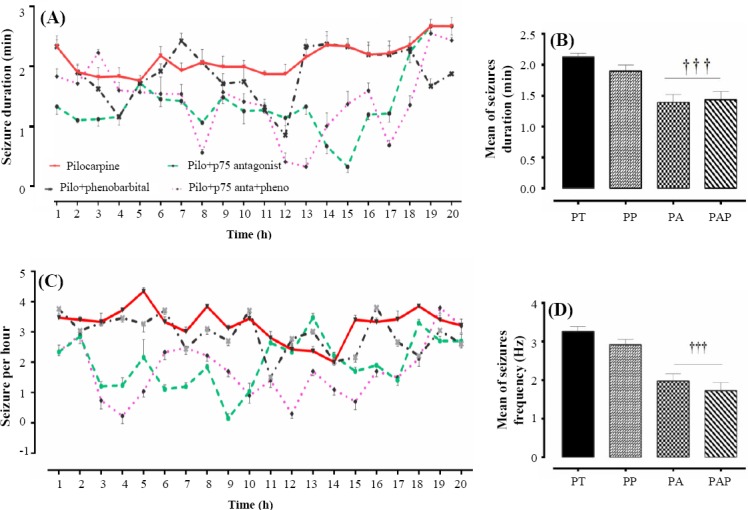
The results of 20-hour video monitoring of all groups indicating changes in the duration (A) and frequency (C) of spontaneous recurrent seizures. Histograms (B and D) indicate the average (± SEM) of duration and frequency of four different experimental groups. PT, pilo-treated; PP, pilo-pheno; PA, pilo-p75 anta; PAP, pilo-p75 anta + pheno

Two weeks after SE induction, continuous 20 hours video monitoring was used, and the average of frequency and duration of SRS was investigated following the blockade of p75NTR. The results showed that p75NTR blockade by antagonist injection alone or combined with phenobarbital markedly reduced the frequency and duration of the recurrent seizures, whereas phenobarbital treatment alone had no significant effect on either the frequency or duration of SRS (Fig. 2).

### Anxiety-related behaviors induced by pilocarpine

**Fig. 3:**
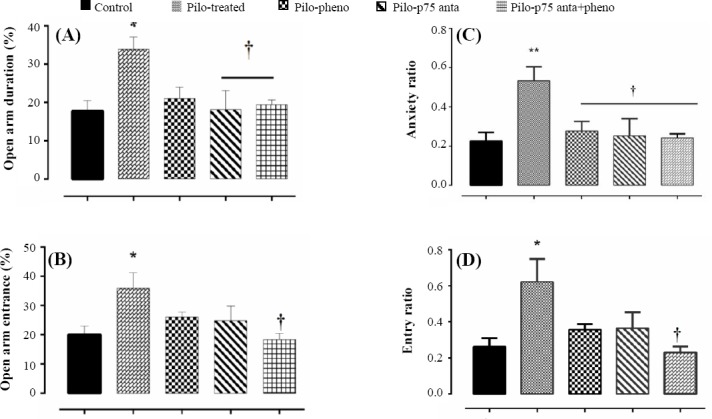
Anxiety-related behaviors of pilocarpine-treated rats and the controls in the elevated plus-maze test. Behavioral testing was performed two weeks after the SE induction. (A) The percentage of time that the rats spent in the open arm; (B) the percentage of entrance of rats in the aversive open arms of the maze; (C) the anxiety and (D) entry ratios in different experimental conditions. ^*^indicates the statistical difference from the control group (^*^p < 0.05; ^**^p < 0.01) and †shows values different from the epileptic rats.

In elevated plus maze test, the open-arm entrance and time spending on the open arms of the maze were considered as a measure of the stress level[43]. In the epileptic rats, the percentage of time spent in open arm was significantly longer (33.88 ± 3.24; *p* < 0.05) compared to control rats (17.8 ± 2.7; Fig. 3A). In addition, a significant increase in the percentage of entries in open arm was observed in the epileptic rats (35.9 ± 5.2; *p* < 0.05) when compared to the controls (20.01 ± 2.9; Fig. 3B). Treatment with p75NTR antagonist alone or combined with phenobarbital had a significant shortening effect on the percentage of time spent in the open arm compared with pilocarpine-treated alone rats (18.11 ± 4.9 and 19.4 ± 1.3, for pilo + p75 antagonist and pilo + p75 antagonist + pheno; *p* < 0.05; Fig. 3A). However, phenobarbital treatment alone had no significant effect on the time spent in open arm compared with the epileptic rats. Also, the treatment of epileptic rats with p75 antagonist (24.8 ± 4.9) or phenobarbital alone (26.04 ± 1.7), but not in combination with phenobarbital (18.39 ± 2, *p* < 0.05), had no significant effect on the percentage of open arm entries (Fig. 3B) when compared to the epileptic alone rats.

The anxiety ratio, which was calculated by dividing the percentage of time spent in open arm by the percentage of time spent in closed arm, was significantly higher in pilocarpine-treated rats (0.53 ± 0.07 versus 0.23 ± 0.04 in control rats; *p* < 0.01; Fig. 3C). In addition, entry ratio (percentage of open arm entry/percentage of closed entry) was significantly higher in epileptic rats (0.62 ± 0.12 versus 0.26 ± 0.04 in control group, *p* < 0.05; Fig. 3D).

Treatment with phenobarbital or p75 antagonist alone or combined treatment with p75 antagonist and phenobarbital significantly reduced the anxiety ratio (*p* < 0.05). However, only the decreasing effect of combined treatment with P75 antagonist and phenobarbital on entry ratio was statistically significant (0.23 ± 0.03, *p* < 0.05; Fig. 3D), when compared to epileptic rats.

### Changes in mRNA expression of p75NTR and pro-NGF in the hippocampus

**Fig. 4:**
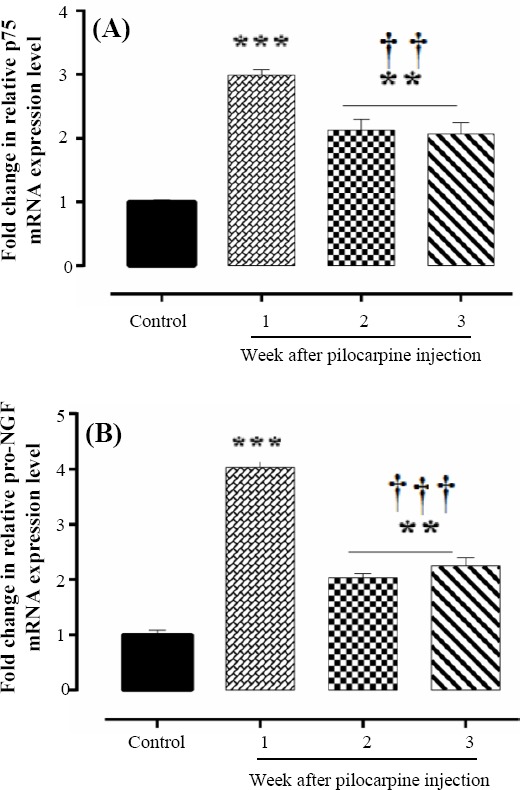
The levels of mRNA expression of p75NTR and pro-NGF in the hippocampus of control and pilocapine-treated animals. (A) Data analysis showed significantly increase in p75NTR expression in pilocarpine-induced SE group at one week (^***^p < 0.001), two weeks, and three weeks (^**^p < 0.01) after pilocarpine injection compared to the control group. Its mRNA expression however was decreased at 2^nd^ and 3^rd^ weeks after pilocarpine-injection (^††^*p* < 0.01) compared to the first week. (B) The pro-NGF mRNA expression was significantly changed at one (^***^p < 0.001), two, and three weeks (^**^p < 0.01) after SE induction compared to the control group. However, its mRNA expression was significantly reduced at 2^nd^ and 3^rd^ weeks, when compared to the first week of SE induction (^†††^*p* < 0.001). The mRNA expressions of p75NTR and pro-NGF were normalized to GAPDH and were expressed as mean ± SD, n = 4.

In the present work, the p75 receptor mRNA expression level was examined at one week, two weeks, and three weeks after induction of SE model. The results showed that the p75NTR expression was significantly higher in pilocarpine-induced epileptic model rats compared with that was seen in control group and reached to its highest level (2.9 ± 0.1, *p* < 0.001) in the first week after the induction of model and then decreased at the second (2.1 ± 0.2) and the third weeks (2.07 ± 0.1) after SE induction. However, it was significantly higher than the control rats at all those time points (1 ± 0.06; Fig. 4A). Next, the pro-NGF expression was examined after epileptic model induction. Interestingly, a similar expression pattern to the p7NTR was observed for pro-NGF mRNA expression in SE group. Within the first week after the induction of epileptic model, pro-NGF gene expression showed an initial significant increase at the first week (4.03 ± 0.2) when compared to the control (1 ± 0.081), and thereafter a decline in the expression was observed at the second (2.033 ± 0.08) and third (2.24 ± 0.1) weeks after SE induction (Fig. 4B). However, the expression level was still significantly higher than that of control group.

## DISCUSSION

In line with previous investigations in epileptic rats[42,44-46], we demonstrated here the up-regulation of mRNA expressions of p75NTR and pro-NGF in a rat pilocarpine-induced SE model, which in turn is associated with the elevated level of aggressive behavior.

Evidence has implied the role of p75NTR receptor and its ligands in the regulation and modulation of neurological and behavioral functions[47-49]. Some prior reports have also indicated that epilepsy often shows a comorbidity with behavioral disturbances, including aggression and agitation[12,17,43], and induction of SE by pilocarpine can also provoke seizure-associated behaviors such as aggressive and unsocial responses. However, the open basis of this coincidence is not fully defined.

The present results indicated that pilocarpine- injected rats displayed less anxiety-like behaviors, which is consistent with previous published reports[50-52] that demonstrated SE induction is associated with decreased anxiety in the elevated plus-maze, as evidenced by longer time spent and higher number of entries in arms. Similar to our finding, Detour and colleagues[50] found that in the elevated plus maze, epileptic rats exhibited a significant higher number of entries and the time spent in open arms, suggesting a reduction in the anxiety-related behavior. This reduction has been attributed to the lesions in amygdala and hippocampus[53-55].

Increased expression of p75 neurotrophin receptor in response to seizure has been reported[44]. Increased expression of p75NTR has also been reported in a variety of neurodegenerative diseases, which may be correlated to the cell fate[56]. Here, the onset of seizure attacks was paralleled by changes in anxiety-related behavior, possibly due to the up-regulation of mRNA expression of the p75 receptor, which has a role in epilepsy and neuropsychiatric disorders. Epileptic patients suffer more frequently from psychiatric and behavioral disorders than the general population, which have a negative impact on their life quality[6,57,58]. Hence, the understanding of underlying mechanism is necessary for introduction of effective treatment strategy for behavioral disorders associated with epilepsy.

Neurotrophins and their receptors have been reported to be involved in epileptogenesis using several animal models of epilepsy[59-61]. p75, which is normally expressed during development, has been shown to be re-expressed in adulthood under various pathological conditions, including epilepsy[15,42]. In addition, Volosin *et al*.[44] have suggested a role for proneurotrophins acting through p75NTR in hippocampal cell death after seizure. Several documents have also suggested a pivotal role for neurotrophins and their receptors in various psychiatric disorders such as depression[62-64].

In the present study, we demonstrated that the up-regulation of P75NTR and pro-NGF is associated with behavioral alterations following the induction of SE by pilocarpine, which is consistent with previous studies[35,36]. It has been shown that neurotrophin receptors, especially P75NTR, play an important role in epileptogenesis and are up-regulated in the animal model of epilepsy and epileptic patients[65,66]. Therefore, we asked whether the blockade of p75 neurotrophin receptor may be a useful therapeutic target for the treatment of behavioral changes associated with epilepsy following SE induction. Our findings for the first time showed that i.p. injection of p75 antagonist (1 mg/kg), alone or combined with phenobarbital (30 mg/kg), significantly reduced the duration and the frequency of SRS. Furthermore, the blockade of p75NTR induced anxiogenic behavior in rats, evidenced by the decrease in the time spent in open arms and the decreased anxiety scores and entry ratio when compared with pilocarpine-treated rats in the elevated plus maze. Moreover, p75NTR inhibition attenuated the behavioral hyperexcitability, as indicated by lower scores in approach- and touch-response as well as finger-snap and pick-up tests.

In summary, our results suggest that the p75NTR blockade attenuates aggressive behavior in epileptic rats, suggesting that the overexpression of p75NTR may be a possible mechanism of the development of aggression and seizures; therefore, its inhibition could be a potential therapeutic approach for psychiatric behaviors associated with epilepsy.
